# Harnessing Oxidative Stress as an Innovative Target for Cancer Therapy

**DOI:** 10.1155/2018/6135739

**Published:** 2018-05-24

**Authors:** Lynne Postovit, Christian Widmann, Peng Huang, Spencer B. Gibson

**Affiliations:** ^1^Department of Oncology, University of Alberta, Edmonton, AB, Canada; ^2^Department of Physiology, University of Lausanne, Lausanne, Switzerland; ^3^Department of Translational Molecular Pathology, University of Texas MD Anderson Cancer Center, Houston, TX, USA; ^4^Department of Biochemistry and Medical Genetics, University of Manitoba, Winnipeg, MB, Canada; ^5^Research Institute in Oncology and Hematology, CancerCare Manitoba, Winnipeg, MB, Canada

One of the hallmarks of cancer is the deregulation of cellular energetics [1]. This allows cancer cells to proliferate and survive in microenvironments that would lead to the death of a normal cell. As a consequence of deregulated cellular energetics, cells produce higher levels of reactive oxygen species (ROS) concomitant with alterations in antioxidant pathways [2]. ROS contributes to cell survival, proliferation, and metastasis in a variety of cancers but left uncontrolled leads to cell death [3]. Cancer cells have adapted to this oxidative stress through various mechanisms allowing them to survive in hypoxia and to become drug resistant [4]. This has given rise to different treatment strategies aiming to enhance ROS production in cancer cells leading to the selective killing of these cells when compared to normal cells.

This special issue on harnessing oxidative stress as an innovative target for cancer therapy focuses both on understanding how cancer cells alter their antioxidant signaling pathways causing drug resistance as well as utilizing alterations in ROS regulation to target cancer cells with novel treatment strategies.

The manuscripts submitted to this special issue were reviewed by at least 2 external reviewers and one guest editor. All the papers were selected on the basis of scientific significance, relevance to topic of oxidative stress and novelty. Mentioned below are the highlights of the manuscripts published in this special issue.

Adaptation to oxidative stress leads to drug resistance [2]. C. Glorieux et al. have shown that chromatin remodeling of the catalase gene leads to increased catalase activation in breast cancer cell lines. The increased expression appeared to be independent of the activation of DNA damage signaling, blockage of protein degradation, or increased mRNA stability. Other redox regulation proteins are also increased in breast cancer. N. Roininen et al. have shown that redox-regulating proteins Nrf2, Keap1, Trx, and Prx1 were increased in breast cancer patients undergoing neoadjuvant chemotherapy. Before chemotherapy, the lower expression levels of these redox regulatory proteins in breast tumors correlated with reduced disease-free survival of patients. This suggests that redox-regulating enzymes might be able to prognosticate cancers and that they may also be excellent targets for treatment.

In addition to drug resistance, cytotoxicity of chemotherapy on normal tissue in patients is a major limitation in treatments [5]. It has been suggested that oxygen pretreatment of tissue could reduce cisplatin cytotoxicity in renal tubular cells of the kidney [6]. However, B. Rasoulian et al. have demonstrated that oxygen pretreatment also reduces the cytotoxicity of cisplatin in malignant cells suggesting that oxygen pretreatment might not be a successful strategy to reduce toxicity in patients treated with cisplatin.

Hypoxia is a poor prognostic factor in cancer, and adaptations to low oxygen levels help drive tumor progression, causing cancer cells to become more aggressive and resistant to chemotherapy [7]. A major mechanism for this adaptation involves the stabilization and activation of the transcription factor, HIF-1. However, recent studies suggest that the regulation of mRNA translation may also play an important role in this process [8]. Indeed, some alterations in translation may occur via an elF4E2-depedendent pathway, leading to increased migration, invasion, and tumor growth. G. Melanson et al. have reviewed the potential for elF4E2 inhibitors to be an effective therapy for hypoxic tumors.

Adaptations to oxidative stress are being explored as novel treatment targets in cancer [2]. Two review articles by S. R. Chowdhury and V. Banerji and R. F. Dielschneider et al. have illustrated that targeting mitochondrial bioenergetics and alterations in lysosomes is an effective strategy to selectively kill cancer cells. Both of these targets in cancer cells utilize elevated ROS levels to induce cell death.

Novel drugs are also being investigated to induce oxidative stress in cancer cells leading to cell death. L. Wang et al. showed that the combination of triethylenetetramine and ascorbic acid leads to synergistic cell death mediated by elevated levels of hydrogen peroxide. Similarly, L. D. Santos et al. showed that xylopine (an aporphine alkaloid agent) induced apoptosis dependent upon increased ROS levels in cancer cells. Using an inhibitor of the redox-regulating protein Nrf2, M. Wang et al. showed that this inhibitor effectively induces apoptosis in combination with UVA irradiation and reduced tumor growth in mouse models. These innovative drugs could provide novel treatment strategies to target ROS adaptations in cancer cells leading to cell death while sparing normal cells.

Novel drugs given as monotherapies are unlikely to be effective due to cellular adaptations leading to drug resistance. Rational drug combination needs to be developed to combat drug resistance in cancer. G. Carrasco-Torres et al. showed that a combination of maleic anhydride derivatives (prooxidant) and quercetin (antioxidant) could induce cell death in cancer cells but not normal human epithelial cells. This suggests that using drugs that first give an oxidative response followed by an antioxidant drug might be a potential new treatment strategy.

Taken together, this special issue gives insight into the potential of targeting the oxidative stress response in cancer cells that could overcome drug resistance and spare normal tissue. This will provide patients with more therapeutic strategies to combat cancer utilizing the cancers' alterations in the redox defense systems. This will hopefully lead to longer disease-free survival of the cancer patient.

*Lynne Postovit* *Christian Widmann* *Peng Huang* *Spencer B. Gibson*
